# Bereaved Parents' Perspectives on Quality Pediatric End‐of‐Life Care: A Scoping Review

**DOI:** 10.1002/cesm.70103

**Published:** 2026-08-24

**Authors:** Rawnaq Almahadeen, Seilin Uhm, David Wright, Alison Richardson, Richard Wagland, Katherine Hunt, Anne‐Sophie Darlington

**Affiliations:** ^1^ Head of Child and Maternity Health Nursing Department Mu'tah University Al‐Karak Jordan; ^2^ School of Health Sciences, Faculty of Environmental and Life Sciences University of Southampton Southampton UK; ^3^ Cochrane Priority Setting Methods Group Plymouth UK; ^4^ Clinical Professor of Cancer Nursing & End of Life Care University of Southampton & University Hospital Southampton Southampton UK; ^5^ Midwifery and Health, School of Health Sciences University of Southampton Southampton UK

**Keywords:** bereaved parents, communication, family‐centered care, palliative care, pediatric end‐of‐life care, quality improvement

## Abstract

**Background:**

Pediatric end‐of‐life care profoundly impacts families, yet existing reviews on bereaved parents' experiences lack methodological rigor and comprehensive synthesis. Identifying key quality indicators from parents' perspectives is essential for improving care.

**Objectives:**

This scoping review synthesizes evidence on bereaved parents' views of high‐quality pediatric end‐of‐life care, identifying critical domains and developing a thematic framework.

**Methods:**

We conducted a scoping review adhering to PRISMA‐ScR guidelines. Searches in MEDLINE, CINAHL, PsycINFO, PsycArticles, and AMED included studies without date restrictions. Two reviewers independently screened studies, extracted data, and conducted inductive thematic analysis.

**Results:**

From 4962 records, 20 studies (1997–2015) met inclusion criteria, comprising 16 qualitative and 4 quantitative studies. Three themes emerged: (1) Interpersonal Interaction (compassionate communication, shared decision‐making); (2) Interdisciplinary Care (symptom management, emotional, spiritual, and sibling support); and (3) Practical Support (care accessibility, skilled staff, financial/logistical assistance).

**Conclusions:**

Bereaved parents advocate for a holistic, family‐centred approach integrating empathetic communication, clinical expertise, and systemic support. These findings inform quality improvement initiatives and policy development in pediatric palliative care.

## Summary

- When a child is seriously ill and approaching the end of life, the care provided to the child and family can have a lasting impact on parents' grief and wellbeing.
- This review explored what bereaved parents describe as important in high‐quality paediatric end‐of‐life care. The review included 20 studies published between 1997 and 2015. Most studies were conducted in high‐income countries, including the United States, Australia and Canada. Parents had cared for children with serious life‐limiting conditions, including cancer, neurological conditions and other complex illnesses.
- Across the studies, parents described three main areas of care as especially important. First, they valued honest, clear and compassionate communication from healthcare professionals. Parents wanted information that was understandable and timely, and they wanted to be included in decisions about their child's care.
- Second, parents emphasised the need for care that supported the whole family, not only the child. This included good pain and symptom management, emotional and psychological support, spiritual care where appropriate, support for siblings, and follow‐up care after the child's death.
- Third, parents highlighted practical and system‐level support. Families needed reliable access to services, continuity from staff who knew them and their child, and help with practical pressures such as travel, costs and arranging care at home.
- Overall, the review shows that high‐quality paediatric end‐of‐life care should be compassionate, family‐centred and well‐coordinated. Services should support clear communication, effective symptom management and ongoing care for parents and siblings before and after a child's death. The findings should be interpreted in light of the review's search date, as more recent studies may provide additional evidence.

## 1 Introduction

High‐quality end‐of‐life care for children with life‐limiting illnesses is paramount to alleviating suffering and supporting families through an immensely difficult time. A child's death profoundly affects parents and family members, and understanding parents’ perspectives on what constitutes “quality” in pediatric end‐of‐life care is crucial for improving services. Prior research indicates that parents and physicians may prioritize different aspects of care: for example, parents often value honest communication and emotional support over clinical measures like pain control, whereas clinicians may emphasize symptom management. Such disparities highlight the importance of incorporating the family's voice in defining quality of care.

Several literature reviews have attempted to synthesize parents' experiences and needs in pediatric palliative or end‐of‐life care. Notably, Aschenbrenner et al. [1] and Melin‐Johansson et al. [2] reviewed qualitative studies of parents' experiences, and Virdun et al. [3] conducted an integrative review to identify elements of optimal pediatric palliative care. While these reviews provided valuable insights, each had limitations. They included differing sets of primary studies with minimal overlap and reported somewhat fragmented domains of care. For instance, the earlier reviews lacked clarity in search strategy or inclusion criteria, and collectively they did not capture a comprehensive set of relevant studies due to variability in scope. As a result, the existing literature on parents' perspectives has gaps and inconsistencies, indicating the need for a more rigorous and exhaustive synthesis.

This study was undertaken to systematically map and synthesize the evidence on bereaved parents' perspectives of pediatric end‐of‐life care quality. By conducting a scoping review, we sought to identify the range of aspects of care that have been reported as important, and to collate those aspects into an interpretable framework or set of themes. The goal was to inform the development of evaluation tools and guide improvements in pediatric end‐of‐life care practice. Specifically, we aimed to answer: *What do bereaved parents perceive as the key components of high‐quality end‐of‐life care for children?*

## 2 Methods

### 2.1 Design and Protocol

We conducted a scoping review using the Joanna Briggs Institute (JBI) systematic guidelines [4] and followed the PRISMA‐ScR guidelines [5]. No protocol was registered for this scoping review.

### 2.2 Eligibility Criteria

We included studies that:

- Examined bereaved parents' perspectives on pediatric EOL care,
- Addressed care during the EOL phase or immediately after death,
- Took place in healthcare or community settings (e.g., hospitals, hospices, and homes).

Single‐participant studies and case reports were excluded because the aim of this scoping review was to identify recurring elements of care quality across studies and parental accounts, rather than to synthesise individual case narratives. This decision was made to support comparison across studies and the development of a thematic framework based on shared patterns in bereaved parents' perspectives. We excluded studies of sudden child death, such as accidents or acute unexpected events, where there was no identifiable end‐of‐life care period. Only English‐language publications were considered.

### 2.3 Search Strategy

A comprehensive search (August 2021) was conducted in MEDLINE, CINAHL, PsycINFO, PsycArticles, and AMED. Search terms were derived using a Population–Exposure–Outcome framework. We also manually searched reference lists of relevant studies. Duplicates were removed using EndNote X8. The database search was originally conducted in December 2018 and re‐run in August 2021. No further search update was undertaken as part of the revision of this manuscript. The full search strategy, including database‐specific search terms and search dates, is provided in Supporting Information S1: Appendix 2.

### 2.4 Study Selection

Two reviewers independently screened titles/abstracts, followed by full‐text assessment, resolving discrepancies through discussion or a third reviewer. A PRISMA flow diagram documented the selection process.

### 2.5 Data Extraction and Analysis

Data extraction and thematic coding were undertaken by RA, with regular discussion among the review team [6]. Extracted findings relating to parents' perceptions of care quality were coded inductively in NVivo 12. Codes were first generated from the findings and participant quotations reported in the included studies. Similar codes were then grouped into descriptive subthemes, which were reviewed and refined through team discussion. Subthemes were subsequently organised into broader analytical themes that reflected recurring domains of care quality across studies. Disagreements or uncertainties in coding and theme allocation were resolved through discussion, with reference back to the source studies. The final framework was refined iteratively to ensure that it remained grounded in the included studies while also providing a coherent structure for interpreting bereaved parents' perspectives on paediatric end‐of‐life care. Existing quality indicators, including indirect indicators from adult bereavement and palliative care populations, were considered as sensitising context during interpretation but were not used as a priori coding categories.

## 3 Results

### 3.1 Study Selection and Characteristics

**Figure 1:**
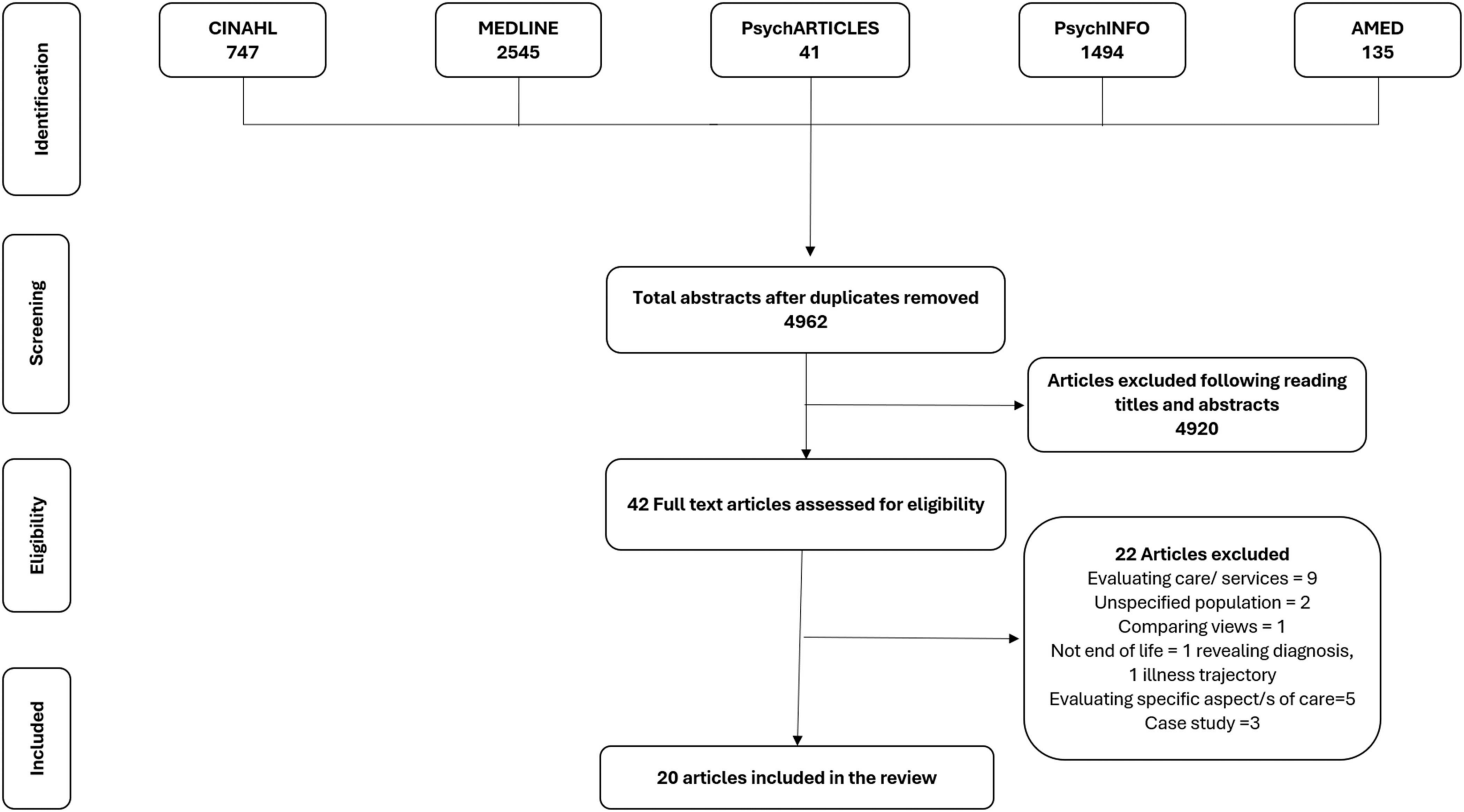
PRISMA flow diagram.

Our search strategy identified 4962 records, which were screened for relevance following de‐duplication. After title and abstract screening, 42 records remained for full‐text review, of which 20 studies met all inclusion criteria and were included in this scoping review. The PRISMA flow diagram (Figure 1) illustrates the study selection process.

The included studies were published between 1997 and 2015 and were conducted across seven countries, primarily in high‐income settings: United States (*n* = 10), Australia (*n* = 4), Canada (*n* = 2), United Kingdom (*n* = 1), Switzerland (*n* = 1), Brazil (*n* = 1), and Malaysia (*n* = 1). All numerical summaries were derived from the study‐level data presented in Table 1. Where demographic, diagnostic or care‐related details were not reported in the original studies, these are recorded as “not reported” and were not included in percentage calculations.

**Table 1:** Characteristics of included studies reporting bereaved parents' perspectives on paediatric end‐of‐life care.

Study	Country	Design	Sample size	Parent characteristics	Child characteristics	Diagnosis	Setting	Care/support described	Notes
James and Johnson [7]	Canada	Qualitative (semi‐structured interviews)	12 parents (8 children)	8 mothers, 4 fathers; 11 married, 1 widowed; Age 35−54 years; Income > $25,000; 10 had college/university education	Mean age at diagnosis 7.6 years; Mean age at death 9.3 years; Mean illness duration 23.8 months; Mean palliative phase 12.9 weeks	Various types of cancer	Home or hospital (Greater Metropolitan Toronto)	Child recognized as special; maintaining normality; caring/connectedness with professionals; retaining parenting responsibility	27 families approached; 11 agreed; 1 excluded; Parents struggled to balance special treatment with normality; Follow‐up care was appreciated
Contro et al. [8]	USA	Qualitative (semi‐structured interviews)	68 family members (44 families)	66% female; 36 mothers, 21 fathers; Mean age 38 years; 74% White, 22% Latino, 4% Asian; 87% English‐speaking, 13% Spanish‐speaking; 51% reported financial burden	44 children; Age range: 16% hours/days, 11% 1−12 month, 23% > 1−5 years, 11% > 5−10 years, 18% > 10−15 years, 20% > 15 years	64% oncologic; 9% cardiac; 9% premature; 18% other	Hospital (Lucile Salter Packard Children's Hospital, Stanford)	Parental involvement; compassionate caregivers; honest communication; sibling support; bereavement follow‐up; pain management	17 died at home; 15 used hospice/home care; Spanish‐speaking families reported isolation and confusion; single events caused lasting distress
Davies and Connaughty [9]	Canada	Quantitative (survey with Likert‐type and descriptive responses)	45 parents	Parents of children who died during a 2‐year period in a regional children's hospital	Not specified in detail	Not specified in detail	Regional children's hospital	Communication skills; staff comfort with death; information provision; small acts of kindness; follow‐up care	Only 20% satisfied with staff communication competence; Only 14% felt adequately informed about deteriorating condition; Parents valued consistency in staffing
Meyer et al. [10]	USA	Quantitative (self‐administered questionnaire)	56 parents (56 households)	64% mothers, 36% fathers; Mean age 42.3 years; 75% married; 91% White; 91% religious affiliation	Children aged newborn to 18 years; Full range of medical/surgical diagnoses; Death after withdrawal of life support	Various medical/surgical diagnoses	3 Boston PICUs (Children's Hospital, Mass General, Tufts‐NEMC)	Adequacy of information; pain management; end‐of‐life decision‐making; social support; religious/spiritual beliefs	58% response rate; 90% physicians initiated withdrawal discussions; 55% felt little/no control; 24% would have made decisions differently; Nurses most involved at death
Seecharan et al. [11]	USA	Quantitative (in‐person interviews)	79 parents/guardians (59 child deaths)	Mothers and female guardians combined for analyses; 19 paired male/female responses; Interviews mean 21.8 months post‐death	Children who died 1998‐2000 at faith‐based teaching hospital	Various (sudden vs anticipated deaths)	Faith‐based teaching hospital	Quality of care (CASC); Grief (TRIG); Pain management; Communication; Bereavement follow‐up	30.6% response rate; 33.2% refused; High satisfaction scores (ceiling effect); Grief scores similar between mothers and fathers; Sudden death mothers had more prolonged grief
Heller and Solomon [12]	USA	Qualitative (telephone interviews)	36 parents (26 women, 10 men)	23 mothers, 10 fathers, 2 grandmothers, 1 aunt; 33 White, 1 Black, 2 Latino; 31 married; Most Christian; Children died 3‐10 months prior	16 boys, 18 girls; 58% aged ≤ 2 years; 44% aged ≤ 1 years; 38% aged ≤ 6 months; 74% died within 1 year of diagnosis	58% genetic/congenital; 15% cancer; 9% trauma; 12% infectious; 3% other	3 US teaching hospitals; 78% died in PICU	Continuity of care; consistent caregivers; informational continuity; relationship building; bereavement follow‐up	78% recalled having ≥ 1 consistent staff person; Continuity synonymous with caring; Lack of continuity led to hypervigilance and mistrust
Maynard et al. [13]	UK	Qualitative (focus groups ‐ ‘parent lunches’)	29 parents (22 families)	8 fathers, 21 mothers; 23 long‐term users (>1 year); 6 bereaved; 2 sites included foster carers/adoptive parents	22 families represented; Children with life‐limiting/threatening conditions	Various life‐limiting/threatening conditions	East Anglia's Children's Hospices (3 residential sites + community)	Communication; family support; liaison with professionals; respite care; transition to adult services; sibling support	8.5% of users participated; Hospice myth needed dispelling; Tension between respite and EOL care; Transition concerns; Foster carers’ needs often overlooked
Meyer et al. [14]	USA	Qualitative (open‐ended questions from questionnaire)	56 parents (same sample as [10])	64% mothers, 36% fathers; Mean age 42.3 years; 75% married; 91% White	Children aged newborn to 18 years; Died after withdrawal of life support	Various medical/surgical diagnoses	3 Boston PICUs	Honest/complete information; ready access to staff; communication/care coordination; emotional expression/support; preservation of parent‐child relationship; faith	Six priorities identified; Information and access were central; Parents valued staff who ‘were real people’; Faith was important for many but some experienced spiritual distress
Monterosso et al. [15]	Australia	Mixed methods (quantitative surveys + qualitative interviews)	Phase 1: 129 parents; Phase 2: 38 parents + 20 service providers	Cancer group (*n* = 19): 68% mothers, 95% good/excellent health, 79% moderate‐severe anxiety; Non‐cancer (*n* = 110): 82% mothers, 61% good/excellent health	Cancer: mean age at diagnosis 6.0 years; Non‐cancer: mean age at diagnosis 1.8 years	Cancer (19); Non‐cancer: 58% severe neurological disability	Western Australia (tertiary centre + community)	Caring impact; financial impact; information needs; carer skills; access to services; coordination	Palliative care poorly understood by health professionals and parents; Non‐cancer parents had more unmet needs; Cancer parents had higher anxiety/depression; Respite use differed significantly between groups
Monterosso and Kristjanson [16]	Australia	Qualitative (semi‐structured interviews)	24 parents	67% mothers; Mean age 43.5 years; 21% fair/poor health; 83% moderate‐severe anxiety; 63% moderate‐severe depression; 53% high financial strain; 21% rural	Mean age at diagnosis 6.9 years; Mean treatment duration 2.7 years; 58% had >16 hospital admissions	42% brain tumour; Others: various cancers	5 Australian tertiary paediatric oncology centres	Understanding of palliative care; realization child was dying; people involved in care; things of greatest importance; open/honest communication; hopes for future families	Parents construed palliative care negatively as ‘cut off’ from hospital; Palliative care = keeping child comfortable; Parents valued home death; Authentic relationships were highly valued
Widger and Picot [17]	Canada	Quantitative (survey ‐ telephone/in‐person)	38 families (39 interviews)	Primarily mothers (only 1 father interviewed separately); 89.7% married/common‐law; 59.5% income $30,000‐60,000; 64.1% had no previous losses	40 children (2 sets of twins); 62.5% male; 32.5% critical care deaths; 37.5% birth unit deaths	27.5% prematurity; 20% malignancy; 17.5% congenital anomaly/syndrome; 17.5% stillbirth; 17.5% other	Eastern Canadian tertiary care centre	Information/communication; relationships; care at death; bereavement follow‐up; pain/symptom management; mementos	65% response rate; 84.6% very satisfied overall; Communication was priority improvement area; Bereavement follow‐up lacking; Mementos important regardless of child's age
Heath et al. [18]	Australia	Quantitative (structured interviews + questionnaires)	96 parents (89 returned questionnaires)	82% female; 95% White; Mean age 43.6 years; 49% > high school education; 60% Christian; Response rate 50%	Mean age at diagnosis 6.7 years; Mean age at death 9.4 years; Mean disease duration 2.7 years	36% leukaemia; 32% brain tumour; 32% other solid tumours	Royal Children's Hospital, Melbourne	Primary oncologist care; home‐care nurses; transition to palliative care; decision‐making; sibling support	67% rated primary oncologist care as ‘very good/excellent’; 63% felt caregivers worked well as team; Pain/suffering did not influence satisfaction ratings
Meert et al. [19]	USA	Qualitative (interviews + focus groups)	Phase 1: 33 parents (26 children); Phase 2: 13 parents (10 children) + 15 chaplains	Phase 1: 64% female, 55% White, 45% Black, 64% married; Phase 2: 69% female, 85% White, 15% Black	Phase 1: median age 4.5 years; 69% chronic condition; 31% acute; Phase 2: median age 15 years; 80% chronic	Phase 1: 31% congenital heart, 19% trauma, 11% malignancy; Phase 2: 50% congenital heart	Midwestern urban PICU	Connection with child; cultural/religious respect; personal/professional support; compassion; trust; honest communication; privacy; sacred atmosphere; bereavement support	Four overarching categories identified: Who I Am, While My Child Was Dying, My Child's Death Context, My Bereavement Journey
Monterosso et al. [20]	Australia	Quantitative (face‐to‐face/telephone questionnaires)	69 parents	71% mothers; 80% married; 75% aged > 41 years; 70% metropolitan; 56% full‐time carer during palliative phase	Mean age at diagnosis 6.6 years; Mean age at death 9.5 years; Mean treatment duration 2.9 years; 62% had ≥ 16 admissions	35% brain/spinal tumours; 10% ALL; 16% neuroblastoma; Others: various	5 Australian tertiary paediatric oncology centres (WA, NSW, QLD)	Hospital services; community services; respite/support; carer needs; service/educational needs; family needs	50% severe anxiety; 42% severe depression; Most valued: pain team, oncologist, OT; 87% relied on immediate family for respite; Parents needed honest information and practical support
Inglin et al. [21]	Switzerland	Qualitative (semi‐structured interviews)	15 parents (all mothers)	German‐speaking parents; Children receiving active palliative care (*n* = 9) or deceased within previous 2 years (*n* = 6)	13 of 15 children had at least one sibling; Age range: >16 years (*n* = 5), > 6‐12 years (*n* = 2), > 12−18 years (*n* = 2)	5 cancer; 5 neurological; 5 other (e.g., cardiac disease)	4 Swiss children's hospitals; 13/15 received home care	Communication; decision‐making; coordination of care; home care support; bereavement care	Parents of non‐cancer children reported lack of psychosocial support; Cancer parents needed better care coordination; Preference for home‐based care; Bereavement support was desired
Weidner et al. [22]	USA	Qualitative (semi‐structured interviews + focus groups)	29 parents (20 families)	70% mothers, 30% fathers; 28 White, 2 African American; Children died 2004‐2005; 8 families received hospice care	Various ages (infants to children); Died from illness, chronic condition, or birth defect	Malignancy; premature birth; cardiac, neurologic, gastrointestinal illnesses	Large Midwestern US paediatric hospital + home hospice	Respect for family's role; comfort; spiritual care; access to care/resources; communication; support for decision‐making; caring/humanism	Seven dimensions identified; 99 families contacted, 10 opted out, 48 unreached, 19 declined; 20 families interviewed; Comfort and communication universally identified
Robert et al. [23]	USA	Qualitative (focus groups)	14 parents (9 families)	Mean age 51 years; 7 mothers, 7 fathers; 10 White, 3 Hispanic, 1 Arab American; All married; All employed (except 2 homemakers); Varied education	Mean age at death 15 years; 6 boys, 3 girls; All > 10 years at death	3 leukaemia; 5 solid tumour; 1 CNS malignancy	Tertiary comprehensive cancer center (MD Anderson)	Standards of care; emotional care; communication; social support; decision‐making; spiritual care; symptom management	36% response rate; Four major themes identified; Standards of care most discussed; Parents valued trusted relationships and personalized accommodation; Social support was critical
Brooten et al. [24]	USA	Qualitative (semi‐structured interviews)	63 parents	46 English‐speaking (35 mothers, 11 fathers); 17 Spanish‐speaking (9 mothers, 8 fathers); Mean age 35.1 years; 33% Black, 27% White, 40% Hispanic; 65% married; 49% income >$25,000	Mean age at death 43.4 months (SD 66.23); 62% had no other living children	25.5% congenital anomalies; 23.4% prematurity; 14.9% head trauma; 10.6% chromosomal abnormalities	NICU/PICU (4 hospitals in South Florida)	Compassionate/sensitive staff; understandable explanations; experienced/competent nurses; providers did everything; parental involvement in decisions	98.4% identified helpful provider actions; 81% identified unhelpful actions; Only 2 of 63 mentioned palliative/hospice care
El Halal et al. [25]	Brazil	Qualitative (semi‐structured interviews)	15 parents (9 children)	10 parents (7 mothers, 3 fathers) completed all stages; Age range 27−45 years; Education: elementary to college; All but 3 employed; Distance from hospital 10‐309 km	9 children; Median PICU LOS 8 days (range 2‐120); Multiple CPR attempts (median 3, range 1‐5)	Mixed: short bowel syndrome, AVB, ARDS, congenital heart disease, neuroblastoma, renal/hepatic failure	Two Brazilian PICUs (Hospital de Clinicas de Porto Alegre; Hospital Sao Lucas)	Quality of communication; parental participation in decision‐making; moments surrounding death; compassionate care	77% of invited parents attended; Communication was low quality; Parents reported lack of privacy and peaceful environment; Nurses more supportive than physicians
Lan and Yun [26]	Malaysia	Qualitative (focus groups + in‐depth interviews)	14 parents (9 children)	9 mothers, 5 fathers; Age range 33−54 years, median 44 years; Majority secondary school education; Mean income RM 3000/month	9 children; Age range 2.3−14 years; 8 cancer, 1 Prader Willi Syndrome	8 cancer (AML, ALL, Ewing's sarcoma, rhabdomyosarcoma); 1 Prader Willi Syndrome	Malacca General Hospital, Malaysia	Symptom control; communication; place of death; key worker; terminal care plan; practicalities after home death	77% preferred home death; Poor symptom control despite morphine prescription; Lack of 24/7 key worker; No terminal care plans; Police issues with home deaths

Most studies employed qualitative methodologies (*n* = 16, 80%), primarily using semi‐structured interviews with bereaved parents. These studies often employed thematic analysis, grounded theory, or phenomenological approaches to explore parental experiences in depth. Four studies (*n* = 4, 20%) used quantitative survey methods to assess parental satisfaction with end‐of‐life care.

Qualitative studies typically included 5–30 participants, while quantitative surveys included 50–200 parents. Participant demographic reporting varied across studies. Where reported, mothers comprised the majority of respondents, while fathers were less frequently represented. Several studies reported predominantly White and English‐speaking samples, although the extent of reporting on ethnicity, language and socioeconomic characteristics varied across the included studies.

### 3.2 Care Settings and Child Diagnoses

Studies examined parental experiences across multiple healthcare settings, including:

- Hospital‐based care (*n* = 12, 60%): Neonatal and paediatric intensive care units (NICU/PICU) and oncology wards.
- Hospice settings (*n* = 4, 20%): Standalone hospices or hospice services integrated with hospitals.
- Home‐based care (*n* = 3, 15%): Parents receiving palliative care services at home.
- Mixed settings (*n* = 1, 5%): Families experiencing care transitions between hospital, hospice, and home.

Regarding the children's conditions, included studies focused on:

- Oncology (*n* = 9, 45%): Cancer was the most commonly studied diagnosis.
- Congenital or neurological disorders (*n* = 5, 25%): Including conditions such as cerebral palsy and muscular dystrophy.
- Cardiac or respiratory conditions (*n* = 3, 15%): Life‐threatening heart or lung diseases.
- Mixed conditions (*n* = 3, 15%): Studies that included children with more than one diagnostic group, such as cancer, congenital anomalies, prematurity, cardiac conditions, neurological conditions, genetic or metabolic disorders, trauma‐related conditions and other complex life‐limiting illnesses.

### 3.3 Thematic Findings

Through inductive thematic analysis, we identified three overarching themes that encapsulate the elements of care that parents perceived as essential for high‐quality paediatric end‐of‐life care. These themes highlight the importance of communication, holistic family support, and structural healthcare factors.

1. Interpersonal interaction

This theme encompasses the relational and communicative aspects of care, which were consistently identified as the most emotionally impactful domain for parents.

- Honest and complete information: Parents valued transparent, timely, and accessible information regarding their child's condition, prognosis, and treatment options. They appreciated clinicians who provided clarity without false reassurance, as uncertainty heightened their distress. Some parents described frustration when information was withheld or overly medicalised, making it difficult to make informed decisions.
- Compassionate dialogue: Parents highlighted the tone, empathy, and attentiveness of healthcare professionals as key indicators of care quality. Meaningful conversations, where clinicians acknowledged parental emotions, reassured them, and offered space for questions, were highly valued. Non‐verbal communication, such as eye contact, sitting down during discussions, and physical gestures of support, was also perceived as significant.
- Family involvement in decision‐making: Parents wanted to be active participants in treatment and care decisions, rather than passive recipients of information. Many described positive experiences when clinicians sought their input, explained options, and involved them in goal‐setting. In contrast, some studies reported parental distress when decisions were made without their consultation, particularly regarding life‐sustaining treatments.
- Trust and relationship building: Continuity in staffing facilitated stronger relationships between families and care providers, enhancing trust and emotional security. Parents described profound appreciation for clinicians who consistently checked in on them, remembered their child's name, and displayed genuine investment in their well‐being. Conversely, frequent staff rotations led to disruptions in rapport, forcing parents to repeatedly explain their situation, which increased frustration.

2. Interdisciplinary care for the child and family

This theme highlights the need for comprehensive, family‐centred support that addresses medical, emotional, and practical aspects of care.

- Symptom management: Pain control was the most frequently cited concern across studies. Parents stressed that uncontrolled symptoms—especially pain and breathlessness—were intolerable and deeply distressing. High‐quality care involved proactive pain management strategies, continuous assessment, and individualised symptom relief plans. Some parents reported dissatisfaction when clinicians were hesitant to escalate pain relief, fearing oversedation.
- Emotional and psychological support: Parents highlighted the need for mental health support for themselves and their children. Grief counselling, peer support groups, and psychosocial interventions were valued but inconsistently provided. Some parents reported feeling isolated in their anticipatory grief, particularly when their wider support network disengaged.
- Sibling support: Parents frequently expressed concern about the well‐being of the child's siblings, who often felt neglected, confused, or anxious. High‐quality care involved age‐appropriate explanations, inclusive hospital visits, and access to sibling support programmes. However, many parents felt that siblings' needs were overlooked, leading to long‐term emotional struggles.
- Bereavement care: Parents highly valued follow‐up contact from healthcare providers after their child's death. Gestures such as condolence letters, phone calls, or invitations to memorial services were seen as deeply meaningful. However, some studies noted that parents felt abandoned after discharge, receiving no bereavement support.

3. Practical support and systems issues

The final theme reflects structural and logistical factors that affected care quality.

- Access to care and continuity: Parents valued 24/7 access to paediatric palliative services, with a single point of contact for urgent concerns. Lack of out‐of‐hours support caused significant distress, particularly for families managing home‐based palliative care.
- Competence and training of healthcare providers: Parents expected clinicians to have specialised paediatric palliative care training, particularly in pain management and end‐of‐life communication. Inadequate training led to suboptimal care, with parents reporting inconsistent guidance or conflicting recommendations between different providers.
- Financial and logistical support: Many parents faced financial hardship, particularly related to travel costs, medical expenses, and time off work. Some received charitable assistance or hospital subsidies, but others struggled to access financial support. Practical challenges such as long hospital commutes or lack of home nursing services added further stress.

Table 2 presents the frequency of key themes across the included studies and provides illustrative quotations or examples for high‐frequency themes, including communication, decision‐making involvement, symptom management, access and continuity of care, and bereavement follow‐up.

**Table 2:** Frequency of themes across reviewed studies.

Theme/sub‐theme	No. of studies (out of 20)	Representative quote/summary
Honest communication	18	“We needed to know the truth, even if it hurt.”
		“Having one constant person throughout was important. He was honest with us, sometimes he'd say, ‘I have no idea,’ but then he'd go and talk to someone and get the answer for us. Having one person follow you throughout is probably the biggest sense of relief” ([8] p.16).
		“There was one number to call when you had problems, and they contacted the person that you needed at that moment…It wasn't like you had 10 numbers…it made it a lot easier for us.” ([22] p.282).
Decision‐making involvement	16	Parents wanted to be part of the team.
		“We received the information of what was to be done or what could be done, without deciding” ([25] p.498)
Sibling support	9	Often overlooked, but deeply valued.
		“They should be allowed as much time together [with the patient] as possible. Even if they don't show it, it affects them. They should be included in discussions. Staff should pay attention to the siblings, too. Get to know them.” ([8] p.17)
Bereavement follow‐up	10	“A condolence card meant the world.”
Symptom Management	17	Pain and breathlessness were top concerns.
		“Towards the end, he suffered a lot…It would have been good if during the terminal phase, a team from the hospital could visit us and ease his discomfort and suffering, at least he could have been satisfied during his last moments, seeing he wanted to go to the hospital to have his pain addressed.” ([26] p.297)
		“We told them she didn't do well on morphine. We saw the pain she was in. For 48 h we kept telling them it wasn't helping. No matter how much morphine they'd give her, she was flopping around on the bed. So we stood there the whole time…she was moaning in pain. [Crying] Those are the images that are the most painful, that she had to suffer. We were helpless.” ([8] p.17).
Spiritual support	5	Religious care not always offered.
		“We have our own coping mechanisms…I think a person's religion plays a huge role…that is what carried us through, having our faith and our church's support.” ([22] p.282).
		“My faith and trust in God, who was in charge of Jessie. Knowing she would not suffer any more when she went to be home to be with the Lord.” ([14] p.652)
Access to care/continuity	12	24/7 access cited as crucial.
		“The inflexibility of the booking system was a key area of dissatisfaction. Parents wanted more ability to negotiate when they could access care.” ([13] p.626).
Financial/logistical strain	6	Hidden costs added to distress.
		“I guess they made you feel that our main concern is our child and being with our child…not coming up with the money for her to be here. The psychologist had contacted my insurance…she had already filled in my insurance company so I didn't have to reiterate the whole situation and try to figure how things were going to work out.” ([22] p.282).

## 4 Discussion

This scoping review synthesised bereaved parents’ perspectives on paediatric end‐of‐life care, identifying three interdependent domains of quality: Interpersonal Interaction, Interdisciplinary Care for the Child and Family, and Practical Support and Systems Issues. These findings reinforce the need for a holistic, family‐centred approach to paediatric palliative care that extends beyond symptom management to encompass emotional, spiritual, and systemic support.

### 4.1 Comparison With Existing Literature

Our findings align with previous reviews of bereaved parents' experiences of paediatric palliative and end‐of‐life care, which have consistently identified communication, emotional support, symptom management and family‐centred care as core elements of care quality [1, 2, 3]. However, our synthesis reorganises these elements into three broader domains: interpersonal interaction, interdisciplinary care for the child and family, and practical support and systems issues. This structure highlights that parents' evaluations of care quality are not limited to clinical management, but are shaped by relational, emotional and organisational dimensions of care. This interpretation was also informed by the development of VOICES‐C, a post‐bereavement questionnaire designed to assess the quality and experiences of paediatric end‐of‐life care across healthcare settings [27].

The geographical concentration of studies in high‐income countries indicates potential underrepresentation of perspectives from low‐ and middle‐income countries (LMICs), where access to paediatric palliative care is often limited [28]. The prevalence of oncology‐focused studies suggests a research bias towards cancer‐related end‐of‐life care, while children with non‐malignant, genetic, metabolic, neurological and rare life‐limiting conditions remain less visible in the evidence base. This is important because paediatric palliative care is relevant across a wide spectrum of life‐limiting conditions, including congenital anomalies, neurological disorders and progressive non‐malignant conditions, not only cancer.

### 4.2 Theoretical Implications

These findings can be understood through the lens of Family Systems Theory [29], which posits that the distress of one family member affects the entire family unit. Bereaved parents consistently reported that effective communication, family involvement in decision‐making, and sibling support were crucial aspects of care. This underscores the need for interventions that do not isolate the child as the sole recipient of care but instead recognise the family as an interconnected system requiring support.

Additionally, the emphasis on trust and communication aligns with attachment theory [30], suggesting that parents who feel emotionally supported by healthcare providers may cope better with their loss. Conversely, negative care experiences—such as feeling excluded from decision‐making—may contribute to complicated grief [31], reinforcing the need for bereavement follow‐up services.

### 4.3 Practical Applications

This review identifies three practical implications for paediatric palliative and end‐of‐life care. First, healthcare providers require structured training in empathetic, clear, and culturally competent communication. Research has shown that communication skills can be improved through simulation‐based training [32], which could be implemented more widely in paediatric palliative care settings. Second, services should develop more consistent bereavement follow‐up. Parents described ongoing contact after the child's death, such as condolence letters, follow‐up calls, memorial events or referral to grief support, as meaningful. A structured approach to bereavement care could help reduce variation between services and ensure that families are not left unsupported after the child's death. Third, paediatric end‐of‐life care should be delivered through an integrated multidisciplinary model. Parents' accounts highlighted the importance of coordinated input from medical and nursing staff, specialist palliative care teams, psychologists, social workers, chaplains and bereavement services. Ensuring timely access to these professionals may improve the continuity of care and better address the emotional, spiritual, practical and clinical needs of the child and family.

### 4.4 Policy Implications

Many parents expressed a desire to care for their child at home but faced challenges due to limited home nursing services and bureaucratic barriers. Policymakers should prioritise funding for community‐based palliative care and develop streamlined pathways for families seeking home‐based support. The financial burden of paediatric end‐of‐life care was a concern for many families. This highlights the need for dedicated paediatric palliative care funds and improved insurance coverage to alleviate economic strain. Institutions could also implement family liaison services to assist with financial aid applications and logistical arrangements.

### 4.5 Cultural Considerations

The predominance of mothers among respondents may limit the extent to which the findings reflect fathers' perspectives on paediatric end‐of‐life care. Similarly, the concentration of studies among White, English‐speaking and high‐income‐country populations limits the transferability of findings to more culturally, linguistically and socioeconomically diverse populations. Future research should prioritise underrepresented parent groups and settings, including fathers, non‐English‐speaking families and families in LMICs.

Although this review included studies from diverse geographical regions, most were conducted in high‐income Western countries. The perspectives of bereaved parents in LMICs remain underrepresented, despite the fact that access to palliative care in these regions is often highly limited [33]. Future research should explore cultural variations in parental experiences and assess how resource constraints impact care quality.

Additionally, studies in culturally diverse settings indicate that spiritual and religious beliefs play a significant role in end‐of‐life experiences. While our review found that parents appreciated the inclusion of chaplaincy services, healthcare providers may need additional training in culturally competent care to better accommodate diverse religious traditions and death rituals.

## 5 Limitations and Future Research

This study has several limitations. First, most included studies were retrospective, meaning that parental accounts may have been influenced by memory biases or grief‐related distress. Future research could employ prospective longitudinal designs to examine how parents' perceptions of care evolve over time. Second, the overrepresentation of mothers in the included studies may limit the generalisability of the findings. Fathers' perspectives on paediatric end‐of‐life care remain understudied, highlighting the need for research that specifically examines paternal grief and involvement in care decisions [34, 35]. Third, the exclusion of non‐English publications may have introduced language bias, limiting insights from non‐Western contexts. Future systematic reviews should incorporate a broader range of multilingual studies to ensure a more globally representative synthesis. Fourth, the search was last run in August 2021, and no further update was undertaken during manuscript revision. Therefore, studies published after this date were not captured. Given ongoing developments in paediatric palliative and end‐of‐life care, the findings should be interpreted as reflecting the evidence available up to August 2021. A further limitation is that bereaved parents and other lived‐experience contributors were not directly involved in developing or validating the thematic framework. Future research should involve bereaved parents, families and other interest‐holders to assess the relevance, acceptability and comprehensiveness of the proposed domains [36].

## 6 Conclusion

Bereaved parents identify high‐quality paediatric end‐of‐life care as compassionate, family‐centred and well‐coordinated. Across the included studies, parents valued honest communication, involvement in decision‐making, effective symptom management, continuity of care, emotional and bereavement support, and practical assistance for the wider family. These findings highlight the need for paediatric palliative care services that integrate clinical expertise with relational, psychological, spiritual and practical support. Future research should update the evidence base and include more diverse perspectives, particularly from fathers, families from LMICs, and bereaved parents as lived‐experience contributors.

## Author Contributions

**Rawnaq Almahadeen:** data curation and investigation and writing – review and editing and project administration and formal analysis. **Seilin Uhm:** writing – review and editing and project administration and writing – original draft and funding acquisition. **David Wright:** investigation and writing – review and editing and project administration and writing – original draft. **Alison Richardson:** conceptualization and supervision. **Richard Wagland:** supervision and writing – review and editing. **Katherine Hunt:** conceptualization and supervision and validation and methodology and writing – review and editing. **Anne‐Sophie Darlington:** conceptualization and supervision and methodology and writing – original draft.

## Conflicts of Interest

The authors declare no conflicts of interest.

## Declaration of Generative AI Use

Generative AI tools were used to support grammar checking and language editing during manuscript preparation. The authors reviewed and approved all AI‐assisted edits and take full responsibility for the content, accuracy and integrity of the final manuscript.

## Supporting information


Supporting File

## Data Availability

The data that support the findings of this study are available from the corresponding author upon reasonable request.
