# Supplementary material for: Bereaved Parents' Perspectives on Quality Pediatric End‐of‐Life Care: A Scoping Review
Source: Cochrane Evid Synth Methods. 2026 Aug 24;4(5):e70103. doi: 10.1002/cesm.70103 (PMC13516995; doi:10.1002/cesm.70103)
Supplement: Supplementary file 1 [file CESM-4-e70103-s001.docx]

# Appendices

## 1. PRISMA-ScR checklist

| Section/Item | Reported in manuscript | Location/Notes |
| --- | --- | --- |
| TITLE | Yes | Title identifies the article as a scoping review. |
| ABSTRACT |  |  |
| Structured summary | Yes | Abstract includes Background, Objectives, Methods, Results and Conclusions. |
| INTRODUCTION |  |  |
| Rationale | Yes | Introduction explains the need for a rigorous synthesis of bereaved parents’ perspectives. |
| Objectives | Yes | Research question stated at the end of the Introduction: “What do bereaved parents perceive as the key components of high-quality end-of-life care for children?” |
| METHODS |  |  |
| Protocol and registration | Yes | Methods states that JBI guidance and PRISMA-ScR were followed, and that no protocol was registered. |
| Eligibility criteria | Yes | Eligibility criteria specify included populations, end-of-life care context, settings, language restrictions, and exclusions for single-participant studies, case reports and sudden deaths without an identifiable end-of-life care period. |
| Information sources | Yes | Search Strategy lists MEDLINE, CINAHL, PsycINFO, PsycArticles and AMED; reference lists were manually searched; searches were conducted in December 2018 and re-run in August 2021. |
| Search | Yes | Manuscript states that the full search strategy, including database-specific search terms and dates, is provided in Appendix 2. |
| Selection of sources of evidence | Yes | Study Selection states that two reviewers independently screened titles/abstracts and full texts, with disagreements resolved by discussion or a third reviewer. |
| Data charting process | Yes | Data extraction and thematic coding are described, including RA’s role and review-team discussion. However, the manuscript does not clearly state whether a data charting form was piloted or whether data extraction was independently checked. |
| Data items | Yes | Data extraction focused on findings relating to parents’ perceptions of care quality; study characteristics are reported in Table 1. |
| Critical appraisal of individual sources of evidence | Not applicable | No formal critical appraisal was undertaken, consistent with the purpose of this scoping review. |
| Synthesis of results | Yes | Data Extraction and Analysis describes inductive thematic analysis in NVivo 12, coding, grouping into subthemes, development of analytical themes, and comparison with existing quality indicators as sensitising context. |
| RESULTS |  |  |
| Selection of sources of evidence | Yes | Results report 4,962 records screened after de-duplication, 42 full-text records assessed and 20 studies included; PRISMA flow diagram is cited. |
| Characteristics of sources of evidence | Yes | Results and Table 1 summarise study year, country, design, sample characteristics, care setting and child diagnoses, where reported. |
| Critical appraisal within sources of evidence | Not applicable | No formal critical appraisal was undertaken and no quality categories are reported. |
| Results of individual sources of evidence | Yes | Table 1 provides study-level characteristics; Table 2 provides themes and illustrative examples/quotations. |
| Synthesis of results | Yes | Results present three overarching themes: interpersonal interaction, interdisciplinary care for the child and family, and practical support and systems issues. |
| DISCUSSION |  |  |
| Summary of evidence | Yes | Discussion summarises the three interdependent domains of care quality and compares findings with previous literature. |
| Limitations | Yes | Limitations include retrospective designs, overrepresentation of mothers, English-language restriction, no search update after August 2021, and lack of lived-experience involvement in framework development. |
| FUNDING |  |  |
| Funding | Yes | Funding statement added: no specific funding was received; SU was supported by the NIHR Pre-Application Support Fund. |

## 2. Search Term Strategy

**A. Generic Boolean search structure**

(pediatric OR paediatric OR adolescent OR child*)AND(palliative OR "end of life" OR hospice OR terminal*)AND(percep* OR view* OR experience* OR need* OR satisfact* OR "quality of care")

**B. CINAHL search strategy**

| Search line | Search terms |
| --- | --- |
| S1 | (MH "Intensive Care Units, Pediatric") OR (MH "Hospitals, Pediatric") OR (MH "Pediatric Units") OR (MH "Childhood Neoplasms") OR (MH "Pediatric Oncology Nursing") OR (MH "Pediatric Cardiology") OR (MH "Pediatric Nurse Practitioners") OR (MH "Child, Hospitalized") OR (MH "Adolescent, Hospitalized") |
| S2 | TI (child* OR pediatric* OR paediatric* OR adolescent*) OR AB (child* OR pediatric* OR paediatric* OR adolescent*) |
| S3 | S1 OR S2 |
| S4 | (MH "Palliative Care") OR (MH "Hospice and Palliative Nursing") OR (MH "Hospice Patients") OR (MH "Hospice Care") OR (MH "Hospices") OR (MH "Terminal Care") OR (MH "Terminally Ill Patients") |
| S5 | TI (end N2 life) OR AB (end N2 life) OR TI (terminal N1 (car* OR illness*)) OR AB (terminal N1 (car* OR illness*)) OR TI (palliative N1 (car* OR service*)) OR AB (palliative N1 (car* OR service*)) OR TI (hospice N1 (car* OR service*)) OR AB (hospice N1 (car* OR service*)) |
| S6 | S4 OR S5 |
| S7 | (MH "Quality of Health Care") OR (MH "Quality of Nursing Care") OR (MH "Quality of Care Research") |
| S8 | TI (quality N2 car*) OR AB (quality N2 car*) |
| S9 | S7 OR S8 |
| S10 | S3 AND S6 AND S9 |

**C.MEDLINE search strategy**

| Search line | Search terms |
| --- | --- |
| 1 | exp Child/ |
| 2 | exp Adolescent/ |
| 3 | Pediatrics/ |
| 4 | Hospitals, Pediatric/ |
| 5 | Intensive Care Units, Pediatric/ |
| 6 | child*.ti,ab. |
| 7 | pediatric*.ti,ab. |
| 8 | paediatric*.ti,ab. |
| 9 | adolescent*.ti,ab. |
| 10 | 1 OR 2 OR 3 OR 4 OR 5 OR 6 OR 7 OR 8 OR 9 |
| 11 | Palliative Care/ |
| 12 | Hospice Care/ |
| 13 | Terminal Care/ |
| 14 | Terminally Ill/ |
| 15 | palliative.ti,ab. |
| 16 | "end of life".ti,ab. |
| 17 | hospice.ti,ab. |
| 18 | terminal*.ti,ab. |
| 19 | 11 OR 12 OR 13 OR 14 OR 15 OR 16 OR 17 OR 18 |
| 20 | Quality of Health Care/ |
| 21 | Patient Satisfaction/ |
| 22 | percep*.ti,ab. |
| 23 | view*.ti,ab. |
| 24 | experience*.ti,ab. |
| 25 | need*.ti,ab. |
| 26 | satisfact*.ti,ab. |
| 27 | "quality of care".ti,ab. |
| 28 | 20 OR 21 OR 22 OR 23 OR 24 OR 25 OR 26 OR 27 |
| 29 | 10 AND 19 AND 28 |

**D. PsycINFO search strategy**

| Search line | Search terms |
| --- | --- |
| 1 | DE "Childhood" |
| 2 | DE "Adolescence" |
| 3 | DE "Pediatrics" |
| 4 | child*.ti,ab. |
| 5 | pediatric*.ti,ab. |
| 6 | paediatric*.ti,ab. |
| 7 | adolescent*.ti,ab. |
| 8 | 1 OR 2 OR 3 OR 4 OR 5 OR 6 OR 7 |
| 9 | DE "Palliative Care" |
| 10 | DE "Hospice" |
| 11 | DE "Terminally Ill Patients" |
| 12 | palliative.ti,ab. |
| 13 | "end of life".ti,ab. |
| 14 | hospice.ti,ab. |
| 15 | terminal*.ti,ab. |
| 16 | 9 OR 10 OR 11 OR 12 OR 13 OR 14 OR 15 |
| 17 | DE "Quality of Care" |
| 18 | DE "Client Satisfaction" |
| 19 | percep*.ti,ab. |
| 20 | view*.ti,ab. |
| 21 | experience*.ti,ab. |
| 22 | need*.ti,ab. |
| 23 | satisfact*.ti,ab. |
| 24 | "quality of care".ti,ab. |
| 25 | 17 OR 18 OR 19 OR 20 OR 21 OR 22 OR 23 OR 24 |
| 26 | 8 AND 16 AND 25 |

**E. AMED search strategy**

| Search line | Search terms |
| --- | --- |
| 1 | child* |
| 2 | pediatric* |
| 3 | paediatric* |
| 4 | adolescent* |
| 5 | 1 OR 2 OR 3 OR 4 |
| 6 | palliative |
| 7 | "end of life" |
| 8 | hospice |
| 9 | terminal* |
| 10 | 6 OR 7 OR 8 OR 9 |
| 11 | percep* |
| 12 | view* |
| 13 | experience* |
| 14 | need* |
| 15 | satisfact* |
| 16 | "quality of care" |
| 17 | 11 OR 12 OR 13 OR 14 OR 15 OR 16 |
| 18 | 5 AND 10 AND 17 |

**F. PsycARTICLES search strategy**

PsycARTICLES was searched using free-text terms because of its narrower journal coverage.

TI (child* OR pediatric* OR paediatric* OR adolescent*)

OR AB (child* OR pediatric* OR paediatric* OR adolescent*)

AND

TI (palliative OR "end of life" OR hospice OR terminal*)

OR AB (palliative OR "end of life" OR hospice OR terminal*)

AND

TI (percep* OR view* OR experience* OR need* OR satisfact* OR "quality of care")

OR AB (percep* OR view* OR experience* OR need* OR satisfact* OR "quality of care")

**Note for reviewers**

Searches were originally conducted in December 2018 and re-run in August 2021. The search was not updated after August 2021; this is acknowledged as a limitation in the manuscript. The CINAHL strategy reflects the most detailed retained database-specific search record. Equivalent concepts were applied across MEDLINE, PsycINFO, PsycARTICLES and AMED, using controlled vocabulary and free-text terms where available.
